# Gastroprotective Effects of the Aqueous Extract from *Taraxacum officinale* in Rats Using Ultrasound, Histology, and Biochemical Analysis

**DOI:** 10.1155/2021/8987232

**Published:** 2021-12-21

**Authors:** Maria Eduarda D. C. Zanatta, Daniela Miorando, Amanda M. Stefller, Nátali Roos, Jackeline Ernetti, Ana Júlia Predebon, Heloísa Lindemann, Aline Mânica, Beatriz M. M. Oliveira, Patrícia Z. Serpa, Lilian Bohnen, Viviane Simomura, Denise B. Gomes, Max Vidal-Gutiérrez, Wagner Vilegas, Luisa M. Silva, Walter A. Roman Junior

**Affiliations:** ^1^Pharmacognosy Laboratory, Community University of Chapecó Region, 89809-900 Chapecó, SC, Brazil; ^2^Postgraduate Program in Health Sciences, Community University of Chapecó Region, 89809-900 Chapecó, SC, Brazil; ^3^Institute of Biosciences, São Paulo State University, Campus Litoral Paulista, 01049-010 São Vicente, SP, Brazil; ^4^Postgraduate Program in Pharmaceutical Sciences, Chemical-Pharmaceutical Research Nucleus (NIQFAR), University of Vale do Itajaí, Itajaí, SC 89809-900, Brazil

## Abstract

*Taraxacum officinale* F.H. Wigg. belonging to the family *Asteraceae* is an edible medicinal plant distributed worldwide. This study aimed to determine the gastroprotective effects of aqueous extract of *T. officinale* (AETo) in rats using ultrasound, histological, and biochemical analyses. In this study, gastric ulceration was induced by ethanol or piroxicam. Rats were then treated with AETo (3, 30, or 300 mg/kg). The area and histological appearance of gastric ulcers were quantified, and histochemical analysis was performed. The activity of AETo on inflammatory and oxidative stress markers was assessed in the ulcerated tissue. In addition, we investigated the thickness of the gastric wall using the ultrasound technique. Moreover, chemical analyses of AETo were performed. In rats with ethanol- or piroxicam-induced ulcers, AETo reduced the ulceration area, elevated mucin level, and the gastroprotective effect was confirmed by histological analysis. The gastroprotective effect was accompanied by increased activities of SOD, CAT, and GST, as well as an increase in GSH level and reduction in MPO activity. Furthermore, AETo reduced the thickness of the gastric wall in rats. Phytochemical analysis of AETo indicated phenolic acids and flavonoids as the main active compounds. In conclusion, the gastroprotective effect of AETo involves reduction in oxidative stress and inflammatory injury and increase in mucin content. This study advances in the elucidation of mechanisms of gastric protection of *T. officinale*, contributes to the prospection of new molecules gastroprotective, and proposes the ultrasonographic analyses as a new gastroprotective assessment tool in preclinical studies.

## 1. Introduction

Peptic ulcer is a disease characterized by the rupture of the protective barrier of the epithelial mucosa of the esophagus, stomach, or duodenum [1]. These lesions can affect the mucosa and other deeper layers of the gastrointestinal wall and damage the muscle tissue, leading to complications such as hemorrhage and perforation [2, 3]. The etiology of gastric ulcers is complex and multifactorial, mainly attributed to an imbalance between protective factors (mucus barrier, cytoprotective prostaglandins, antioxidants, bicarbonate secretion, and appropriate microcirculation) and harmful factors (highly acidic environment in the gastric lumen and pepsin activity) [4].

Furthermore, gastric ulcers may involve exogenous agents such as stress, *Helicobacter pylori* infection, smoking, alcoholism, and prolonged use of nonsteroidal anti-inflammatory drugs (NSAIDs). These aggressive factors can increase the production of reactive oxygen species (ROS) that can promote a strong inflammatory response [5].

Despite the protective barrier provided by the epithelial layer, several agents and pathogens can cause inflammation by activating the epithelium, polymorphonuclear neutrophils, and macrophages to produce inflammatory cytokines and other mediators that contribute further to oxidative stress [6]. In addition, the current antiulcer therapy does not focus on these pathophysiological events. Thus, to investigate new, effective, and safe alternatives, extensive pharmacological studies based on natural products have been conducted [7].

*Taraxacum officinale* F.H. Wigg. belonging to the family *Asteraceae* is an edible medicinal plant distributed worldwide. This species is native to Europe and Asia and is known as dandelion or taraxaco in America [8, 9]. Popularly, the infusion obtained from its leaves is used for the treatment of diabetes and hepatic and gastric disorders, as well as for diuretic and anti-inflammatory effects [9–11].

In the recent decade, several studies have reported anti-inflammatory, antioxidant, choleretic, diuretic, hepatoprotective, and immunostimulatory effects of *T. officinale* [12, 13]. Furthermore, preliminary studies were carried out to evaluate gastroprotection in rats [14]. These biological effects are probably related to the presence of sesquiterpenolactones, triterpenoids, steroids, flavonoids, phenolic acids, coumarins, phenols, and saponins in the extracts [15, 16]. However, despite the widespread popular use and large pharmacological potential, the gastroprotective activity of extracts that mimic popular use still needs to be validated.

In pharmacological screening, most experiments are carried out in rodents, and the measurement of gastric lesions is performed after dissecting the stomach. Ulcer lesions can be examined macroscopically and microscopically [17]. However, these techniques used for preclinical evaluation of natural products, for example, can be improved to more accurately assess the inflammatory process at the gastric wall. In this context, to imitate the popular use of *T. officinale*, this study aims to demonstrate the gastroprotective effects of the aqueous extract of the plant in rats and also contribute to the mechanisms underlying this activity, employing the ultrasonography of the gastric wall in parallel to macroscopic and microscopic records, as well as biochemistry analyses.

## 2. Methods

### 2.1. Plant Material

The aerial parts of *T. officinale* were collected in Coronel Freitas (SC), Brazil (26° 54′23″ S, 52° 41′59″ W), in October of 2019. The plant was identified by Prof. Adriano Dias de Oliveira curator of the Herbarium of the Community University of the Region of Chapecó (Unochapecó), where a voucher was deposited (#4865).

### 2.2. Preparation of Extract

The sample of *T. officinale* was dried at 25 ± 5°C and powdered in a knife mill (Ciemlab®, CE430). The aqueous extract (AETo) was obtained by infusion to mimic popular preparations [18]. Thus, we used dry-milled leaves of the plant (100 g) in distilled water at 100°C (1 : 20, w/v) for 10 min The AETo was filtered using a Büchner funnel and concentrated by evaporation under reduced pressure (40°C), lyophilized, and weighed (4.250 g; 4.25% yield).

### 2.3. Mass Spectrometry Analysis

The direct flow infusion of the AETo was performed on Thermo LTQ-XL apparatus (IT-MS) equipped with electrospray ionization (ESI) source, in negative mode, capillary tube at 280°C, spray voltage of 5.00 kV, capillary voltage of –35 V, tube lens of –100 V, and a 10 *μ*l/min flow sample.

Fragmentations (MS/MS) of the samples were performed using the collision-induced decomposition (CID) method against Argon for ion activation. The first event was a full scan mass spectrum to acquire data on ions at the range of *m/z* 1540–2000. The second scan event was an MS/MS experiment performed using a data-dependent scan on the [M-H]^–^ molecules from the compounds of interest at a collision gas flow rate of 30%.

### 2.4. Animals

Female rats of the Wistar strain, 3 months old, weighing between 180 and 200 g, were provided by Central Animal House of Unochapecó and maintained in propylene cages under standard laboratory conditions (12 h light/dark cycle, the temperature of 22 ± 2°C), with free access to food and water. Food was withdrawn 12 h before the experiments, and water was provided ad libitum. All the experiments were performed after approval by the Institutional Animal Ethics Committee of Unochapecó (protocol number 030/2019).

### 2.5. Dose-Response Study

In the traditional use, *T. officinale* is prepared and taken orally in form of infusion with 3.0 g (one teaspoon) of dry leaves in 150 ml of water, drunk three times a day, totaling 450 ml/day [18]. The dry residue in this infusion was equivalent to 0.48%. Thus, there is about 2.160 mg in dry residue in 450 ml. For a person weighing 80 kg, this administration represents an intake of approximately 27 mg/kg/day. Consequently, AETo was tested at 3, 30, and 300 mg/kg, ensuring appropriate doses on a log scale to verify a possible dose-response efficacy properly and achieve a higher dose and a lower dose.

### 2.6. Gastroprotective Activity

#### 2.6.1. Evaluation of Ethanol-Induced Gastric Ulcer

The experiment was conducted according to the methods prescribed in the studies by Morimoto et al. [19] and Al-Sayed, and El-Naga [20]. All doses were based on those described by Boeing et al. [21]. Briefly, after 12 h of fasting, rats (*n* = 8 per group) were pretreated with vehicle (Veh, water plus 1% Tween 80; negative control), carbenoxolone (CBX, 200 mg/kg; positive control), or AETo (3, 30, or 300 mg/kg plus 1% Tween 80) 1 h before administration of 98% ethanol (0.5 mL/100 g) to induce gastric ulcer. There was an additional naive (N) group that did not receive ethanol induction and was treated with distilled water. All treatments were administered intragastrically (1 mL/1000 g, p.o.). After 1 h, the animals were anesthetized (thiopental, 40 mg/kg, i.p.) for ultrasonography [22], and later euthanized (thiopental, 140 mg/kg, i.p.). The stomachs were removed and opened along the greater curvature, and the lesion area (mm^2^) was measured using the EARPs software®.

#### 2.6.2. Evaluation of Piroxicam-Induced Ulcer

The experiment was carried out according to the method proposed by Nwafor et al. [23], with minimal modifications. The rats were randomly divided into five groups (*n* = 8), and previously received orally (p.o) the following treatments: vehicle (Veh, water plus 1% Tween 80), carbenoxolone (CBX, 200 mg/kg), or AETo (3, 30 or 300 mg/kg). There was an additional naive (N) group that did not receive piroxicam induction and was treated with distilled water. After one hour of the treatments, all animals received the piroxicam (100 mg/kg, p.o) [24]. After 6 h, the animals were anesthetized for ultrasonography and then, euthanized. The stomachs were removed and opened along the greatest curvature. Lesions were quantified in each stomach, expressed in square millimeters using the EARP^®^ program.

#### 2.6.3. Sonographic Examination of the Stomach Wall

After anesthesia (thiopental 40 mg/kg), rats were placed in the left lateral decubitus position and subjected to the abdominal ultrasonographic examination to evaluate the stomach wall. Images were recorded after 1 h and 6 h of animal exposure to ulcerative agents (ethanol and piroxicam, respectively) using Esaote model MyLab Delta ultrasonographic equipment with a high frequency (10–22 MHz) model SL 3116 micro linear probe. The thickness of the gastric wall close to the area of injury was measured in centimeters [22, 25].

#### 2.6.4. Histological and Histochemical Evaluation

The samples of ulcerated tissue were fixed in a solution composed of 85%, ethanol 80%, 10% formaldehyde, and 5% acetic acid (v/v) for 24 h [26]. Later, the tissues were dehydrated with alcohol and xylene, embedded in paraffin wax, sectioned at 5 µm, and stained with hematoxylin/eosin (HE). The slides also underwent Periodic Acid Schiff (PAS) staining to quantify mucin levels in pink (strong pink) [27].

### 2.7. Biochemical Analysis of Gastric Tissue

The stomachs were homogenized in 200 mM phosphate buffer (pH 6.5), and the concentration of reduced glutathione (GSH) and lipid hydroperoxides (LOOH) was immediately determined. The remaining homogenate was centrifuged at 11,000 rpm for 20 min at 4°C, and the supernatant obtained was used to measure the activity of superoxide dismutase (SOD), glutathione *S*-transferase (GST), and catalase (CAT). The myeloperoxidase (MPO) activity was determined in the precipitate after centrifugation of homogenate from the stomach's samples.

#### 2.7.1. Evaluation of Oxidative Stress Markers

*(1) Determination of GSH Levels*. The quantification of GSH levels was performed according to Sedlak and Lindsay [28] and Anderson [29]. The homogenate was deproteinized with 12.5% trichloroacetic acid and centrifuged for 15 min at 4,000 rpm. After TRIS buffer (0.4 M, pH 8.9) and 5,5′-dithiobis- (2-nitrobenzoic acid) (0.01 M) were added to the supernatant, and the absorbance at 415 nm was measured. The results were interpolated in a standard curve of GSH (1–10 µg/ml), and results were expressed as *μ*g of GSH/g of tissue.

*(2) Determination of LOOH Levels*. The determination of LOOH levels in the samples of stomachs were determined using Ferrous Oxidation-Xylenol Orange (FOX2) reagent. In this assay, the homogenate was mixed with 90% methanol and centrifuged for 20 min at 11,000 rpm. Thereafter, the supernatant was added to FOX2 reagent composed of 4 mM butylated hydroxytoluene, 250 mM Fe_2_SO_4_, and 25 mM H_2_SO_4_ plus xylenol orange 100 mM and incubated for 30 min at 25°C. After the reaction time, the absorbance at 560 nm was determined, and the results were expressed as mmol/mg of tissue [30].

*(3) Quantification of Superoxide Dismutase (SOD) Activity*. The analysis of the SOD activity was performed using a method to evaluate the ability of a solution to inhibit pyrogallol autoxidation [31]. For this, supernatant aliquots 1 mM pyrogallol was added to 200 mM Tris HCl-EDTA (pH 8.5) and incubated for 20 min. After the reaction time, the absorbance was measured at 405 nm, and the amount of SOD capable of inhibiting the autoxidation of pyrogallol by 50%, relative to the control, was defined as a unit (U) of the SOD activity. The results were expressed as U/mg of protein.

*(4) Determination of the Activity of Catalase (CAT)*. To measure the CAT activity, the reactions were performed in the presence of stomachs supernatant aliquots, 5 mM TRIS/EDTA buffer (pH 8.0), 30% H_2_O_2_, and ultrapure water. The decrease in absorbance was measured at 240 nm for 1 min. The results were expressed in *μ*mol of H_2_O_2_ consumed/min/mg of protein [32].

*(5) Determination of Glutathione S-Transferase (GST) Activity*. To evaluate the enzymatic activity of GST, the described method by Habig, Pabst, and Jakoby [33] was used. The reactions were performed in the presence of samples of stomachs supernatant added to 1 mM 1-chloro-2,4-dinitrobenzene (CDNB), 1 mM GSH, and 100 mM potassium phosphate buffer (pH 6.5) at 25°C. The conjugation of CDNB with GSH was monitored at 340 nm for 90 s, and the results were expressed in mmol of GSH/min/mg of protein [34].

#### 2.7.2. Evaluation of Inflammatory Parameters

*(1) Determination of Myeloperoxidase (MPO) Activity*. The assay was performed according to Bradley et al. [35] and Krawisz et al. [36]. To quantify the MPO activity, the precipitates obtained from stomachs homogenates were suspended in 500 µL 80 mM potassium phosphate buffer (pH 5.4) containing 0.5% hexadecyltrimethylammonium bromide. Then, the solution was again centrifuged at 11,000 rpm for 20 min at 4°C. The absorbance of the supernatant was analyzed at 620 nm in presence of 7.5% H_2_O_2_ and 3,3′,5,5′-tetramethylbenzidine 18.4 mM. The results were expressed as units of millioptical density (mOD) per milligram of protein.

### 2.8. Statistical Analysis

The parametric data were presented as the means ± standard deviation of means, and the significance was tested by one-way analysis of variance (ANOVA) followed by the Bonferroni test using the software GraphPad version 8.00 for Windows (GraphPad Software, La Jolla, CA, USA). The values were represented by means ± standard error of the means (SEM) and *p* values less than 0.05 (*p* < 0.05) were used as the significance level.

## 3. Results

### 3.1. Phytochemical Analysis

The aqueous extract of *T. officinale* (AETo) used in the present study was analyzed by electrospray ionization-ion trap mass spectrometry (ESI-IT-MS) based on a direct infusion technique. The structures of the 14 polyphenolic compounds were determined based on their MS2 fragmentation patterns and compared with the literature (Table 1) [35]–[37].

### 3.2. Effect of AETo on Ethanol-Induced Gastric Ulcers

**Figure 1:**
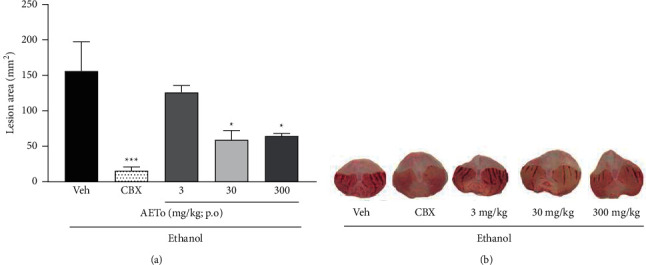
(a) Effect of the aqueous extract from *Taraxacum officinale* (AETo, 3–300 mg/kg) and carbenoxolone (CBX; 200 mg/kg) on the ethanol-induced ulcer (mean ± SEM; *n* = 8). Group induced with ethanol and treated with saline (Veh). One-way ANOVA followed by Bonferroni's test.  ^*∗*^*p* < 0.05 and  ^*∗*^ ^*∗*^ ^*∗*^*p* < 0.001 compared to the vehicle group (Veh). (b) Representatives of macroscopic images from each group.

The rats with ethanol-induced ulcers and treated with vehicle showed considerable damage to the gastric mucosa. However, those receiving AETo 30 or 300 mg/kg showed a significant reduction in the size of the injured area by 62.1% and 58.7%, respectively, compared with the vehicle-treated rats (*p* < 0.05) (Figure 1(a)). On the contrary, AETo at the lowest dose (3 mg/kg) did not exhibit gastroprotective activity. Representative macroscopic images of the ulcerated tissues from the animals in each group are presented in Figure 1(b).

### 3.3. Effects of AETo on Piroxicam-Induced Gastric Ulcers

**Figure 2:**
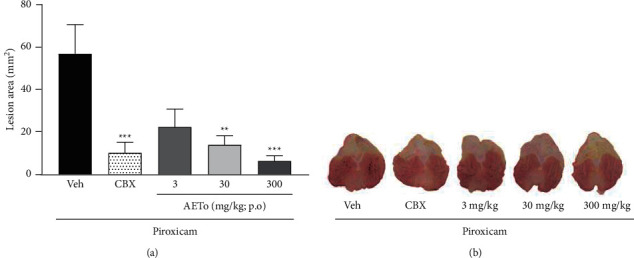
(a) Effect of the aqueous extract from *Taraxacum officinale* (AETo, 3, 30 or 300 mg/kg) and carbenoxolone (CBX; 200 mg/kg) on the piroxicam-induced ulcer (mean ± SEM; *n* = 8). Group induced with ethanol and treated with saline (Veh). One-way ANOVA followed by Bonferroni's test.  ^*∗*^ ^*∗*^*p* < 0.01 and  ^*∗*^ ^*∗*^ ^*∗*^*p* < 0.001 compared to the vehicle group (Veh). (b) Representatives of macroscopic images from each group.

As expected, rats with piroxicam-induced ulcers and treated with vehicle (Veh) revealed a significant injury to the gastric mucosa although AETo at 30 or 300 mg/kg showed a reduction in lesion areas of 75.4 (*p* < 0.01) and 88.8% (both *p* < 0.001), respectively, when compared to the Veh group. Interestingly, the gastroprotection observed for AETo 300 mg/kg was similar to CBX (Figure 2(a)). A macroscopic image of the stomachs of each group, reinforcing the gastroprotective effect of AETo (3, 30, or 300 mg/kg), is shown in Figure 2(b).

### 3.4. Histological and Histochemical Evaluation of AETo on Ethanol and Piroxicam-Induced Gastric Ulcers

**Figure 3:**
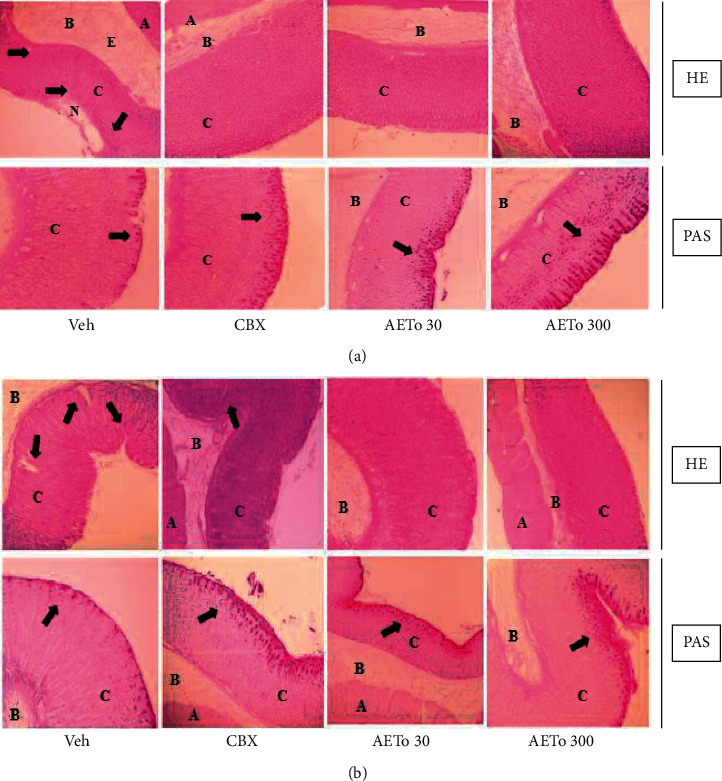
Histological appearance of treatment with the aqueous extract from *Taraxacum officinale* (AETo, 30 or 300 mg/kg) and carbenoxolone (CBX; 200 mg/kg) on the ethanol-induced (a) and piroxicam-induced ulcer (b), after hematoxylin/eosin staining (HE), and after Periodic Acid–Schiff- (PAS-) stained mucin-like glycoproteins. In the images, (A) muscularis, (B) submucosa, (C) mucosa, (N) necrosis, (E) edema. HE: the black arrows in Veh indicate high damage to the gastric mucosa. CBX: AETo 30 and AETo 300 represent gastric mucosa integrity. PAS: Veh had no accumulation of the magenta color in the mucosal cell layer; CBX, AETo 30, and AETo 300 showed increases in PAS staining intensity in the mucosal cells layer compared to the ulcerated group, independent of dose. The black arrow indicates the PAS staining of glycoprotein.

**Figure 4:**
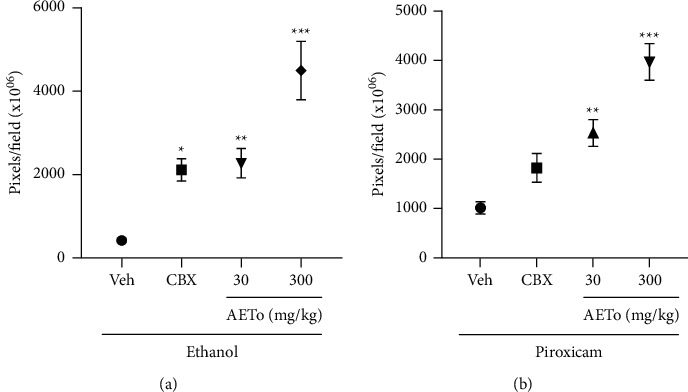
Quantification of PAS-stained mucin-like glycoproteins for the aqueous extract from *Taraxacum officinale* (AETo, 30 or 300 mg/kg) and carbenoxolone (CBX; 200 mg/kg) on the ethanol and piroxicam-induced ulcer (mean ± SEM; *n* = 8). One-way ANOVA followed by Bonferroni's test.  ^*∗*^*p* < 0.05,  ^*∗*^ ^*∗*^*p* < 0.01, and  ^*∗*^ ^*∗*^ ^*∗*^*p* < 0.001 compared with vehicle ulcerated (Veh) group.

Figure 3 shows representative images of histological sections of gastric mucosa stained with hematoxylin/eosin and Periodic Acid-Schiff. These reveal that ethanol-ulcerated stomachs presented had necrotic lesions in the deep gastric mucosa that demonstrated extensive leucocyte infiltration and edema of the submucosal layer. However, the stomach mucosa treated with AETo (30 and 300 mg/kg) or CBX was characterized by preservation of gastric mucosa and an elevation in mucin content compared to the vehicle group (*p* < 0.01, *p* < 0.001, and *p* < 0.05, respectively). In stomachs ulcerated with piroxicam and treated with AETo (30 and 300 mg/kg), there was high preservation of the gastric mucosa, indicated by the accumulation of the magenta color in the mucosal cell layer, representing an increase in mucin content compared to vehicle (*p* < 0.01 and *p* < 0.001, respectively). Interestingly, the piroxicam-induced ulcer group treated with CBX had no mucosal protective effect compared to the vehicle (Figure 4).

### 3.5. Effect of AETo on the Gastric Wall Assessed by Abdominal Ultrasound Examination

**Figure 5:**
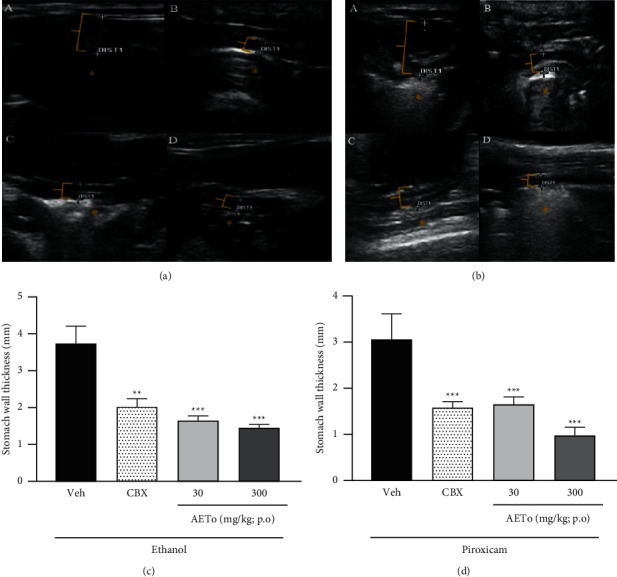
Abdominal ultrasound images of stomachs with ulcers induced by ethanol (a) and piroxicam (b). Effect of aqueous extract of *Taraxacum officinale* (AETo, 30 and 300 mg/kg) on gastric wall spacing of rats with ulcers induced by ethanol (1) and piroxicam (2). Vehicle (Veh), carbenoxolone (CBX, 200 mg/kg). One-way ANOVA, followed by Bonferroni's test,  ^*∗*^ ^*∗*^*p* < 0.01 and  ^*∗*^ ^*∗*^ ^*∗*^*p* < 0.001 compared to the Veh group (mean ± SEM; *n* = 8).

Animals exposed to ethanol or piroxicam showed an increase in gastric wall thickness due to edema caused by direct tissue aggression or indirectly through inflammatory mediators or oxidative stress. In the Veh group, there was a significant increase in the thickness of the stomach wall (3.7 ± 0.04 mm). On the contrary, the groups treated with AETo (30 or 300 mg/kg) showed a reduction in wall thickness by 56.2% and 61.3%, respectively, compared to the Veh group (*p* < 0.01 and *p* < 0.001, respectively). Similarly, CBX administration reduced the thickness by 45.9% (Figures 5(a) and 5(c)).

In addition, in animals with piroxicam-induced ulcers (Veh group), there was an increase in the thickness of the stomach wall, which was decreased by 48.5% after CBX administration. The groups treated with AETo (30 or 300 mg/kg) showed a reduction in thickness by 46.2% (*p* < 0.001) and 68.2% (*p* < 0.001), respectively, compared to the Veh group (Figures 5(b) and 5(d)).

### 3.6. Effect of AETo on the Level of Reduced Glutathione (GSH) and Lipid Hydroperoxides (LOOH)

Table 2 shows that the ethanol ulcerated mucosa showed the levels of GSH reduced by 58.4%, compared to the naive group (1340 ± 13.40 µg/mg of tissue). However, the treatment with AETo (300 mg/kg) was able to avoid the depletion of the GSH levels, promoting an increase of 56.3%, compared to the Veh group, surpassing the activity of the CBX group (1030 ± 18.40 µg/mg of tissue). Similarly, the piroxicam ulcerated group and treated with vehicle group showed a decrease in 35% of the GSH levels compared to the naive (1310 ± 16.9 µg/mg of tissue). However, the administration of AETo and CBX showed no differences in GSH levels when compared to the ulcerated group treated with vehicle (850.6 ± 4.81 µg/mg of tissue).

Regarding the LOOH levels, no differences were observed between the naive (0.18 ± 0.007 mmoL hydroperoxide/mg of tissue) and vehicle groups in either the stomachs of animals ulcerated with ethanol or piroxicam. However, previous administration of AETo (300 mg/kg) in the rats with piroxicam-induced ulceration decreased by 47% of the LOOH levels compared to vehicle (*p* < 0.001) (Table 2).

### 3.7. Effect of AETo on the Activity of Superoxide Dismutase (SOD), Catalase (CAT), and Glutathione-S-Transferase (GST)

As shown in Table 2, the administration of ethanol or piroxicam reduced the SOD activity by 16.5 and 70.6%, respectively, compared to the naive (*p* < 0.001). In the ethanol-ulcerated group, the AETo (300 mg/kg) prevented enzyme depletion compared to vehicle (*p* < 0.05). However, AETo (30 or 300 mg/kg) in the group ulcerated by piroxicam was more effective in preserving the enzymatic damage by 58 and 90% when compared to the vehicle group (*p* < 0.05 and *p* < 0.001).

Compared to the vehicle group, AETo (300 mg/kg) administration in animals ulcerated by ethanol did not increase the GST activity. However, it was able to increase the CAT activity compared to vehicle and naive. Interestingly, in the piroxicam-induced ulcer model, an increase in the GST activity was observed in animals treated with AETo (30 and 300 mg/kg) compared to vehicle (*p* < 0.05 and *p* < 0.001, respectively). Moreover, when compared with the naive group, AETo at a dose of 30 mg/kg prevented depletion and at 300 mg/kg increased the GST activity of GST. In relation to the CAT activity, it did not observe the enzymatic effects with these doses of AETo (Table 2).

### 3.8. Effect of AETo on Myeloperoxidase (MPO) Activity

**Figure 6:**
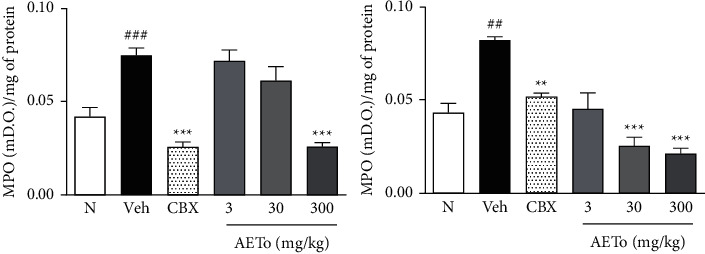
Effects of AETo on MPO levels in stomachs of rats with ethanol- and piroxicam-induced gastric ulcer. (N) (Naive), Vehicle (Veh) ulcer treated with saline, carbenoxolone (CBX, 200 mg/kg). Statistical analysis was performed using one-way ANOVA followed by Bonferroni's test. ^##^*p* < 0.01 and ^###^*p* < 0.001 compared to the naive and  ^*∗*^ ^*∗*^*p* < 0.01 and  ^*∗*^ ^*∗*^ ^*∗*^*p* < 0.001 compared to the vehicle. AETo: aqueous extract from *Taraxacum officinale* (3, 30, or 300 mg/kg).

The vehicle groups ulcerated by ethanol or piroxicam showed a significant increase in the MPO activity by 76.2% and 95,2%, respectively, compared to the naive group (0.074 ± 0.004 and 0.082 ± 0.001 mDO/mg of protein, respectively). In the ethanol-induced ulcer model, the pretreatment with AETo (300 mg/kg) reduced the MPO activity by 66.2%, compared to the vehicle group. In addition, in the piroxicam-induced ulcer model, the pretreatment with AETo (30 or 300 mg/kg) also reduced the activity of this enzyme by 40 and 50%, respectively, when compared to the ulcerated group treated with the vehicle (Figure 6).

## 4. Discussion

Plants of the Asteraceae family are known to have gastroprotective effects [40–42]. Traditionally, the decoction of *T. officinale*, popularly known as dandelion, is used to treat gastric diseases, abdominal pain, and kidney stones, as well as for the treatment of liver disorders due to its hepatoprotective effects [11, 43]. In particular, emerging evidence suggests that *T. officinale* and its constituents have antioxidant and anti-inflammatory activities, resulting in diverse biological effects [12]. However, the gastroprotective activity of the aqueous extract obtained from the plant's leaves, which mimics its popular preparations, has not yet been tested. Therefore, this study provides an analysis of the chemical constituents of *T. officinale* and investigates its gastroprotective property that supports the use of dandelion as a medicinal plant.

The data obtained in this study confirmed the antiulcer potential of the aqueous extract of *T. officinale* (AETo), particularly in the prevention of gastric lesions induced by ethanol and piroxicam, which was confirmed by histological and histochemical analyses. The pharmacological mechanism of gastroprotection by AETo probably involves the role of phenolic compounds identified in the extract, including mucosal defense and activity on inflammatory markers (MPO) and oxidative stress (GSH, LOOH, SOD, CAT, and GST). In addition, a decrease in gastric wall inflammation was observed in ultrasound images.

Ethanol is one of the main etiologic agents of gastric ulcers. It restricts gastric blood flow, leading to hemorrhage and the development of lesions. Another important ulcerogenic mechanism of ethanol is through the production of ROS. These agents promote oxidative stress, increase lipid peroxidation, and decrease antioxidant activity, thus increasing cell damage. This supports the theory that ROS generation and ulcer pathogenesis are closely related [44]. Furthermore, ethanol also induces infiltration of proinflammatory cells and reduces the secretion of protective factors in the mucosa, such as mucus, bicarbonate, and nitric oxide (NO) [45, 46].

In this study, in fact, hemorrhagic lesions were observed in the gastric mucosa of animals exposed to ethanol and pretreated with vehicle (water). However, it was observed that the AETo (30 or 300 mg/kg) administered orally reduced the ulceration area compared to the vehicle and protected the gastric mucosa similar to CBX (positive control). Histological and histochemical analyses of hematoxylin/eosin staining and Periodic Acid–Schiff (PAS) staining confirmed that ethanol decreased the protein concentration, leading to the destruction of the epithelial cells. In contrast, an increase in the glycoprotein content of the gastric mucosa was confirmed by an increase in the intensity of PAS staining in the groups treated with AETo (30 and 300 mg/kg).

In addition, NSAIDs are widely used to treat cardiovascular diseases and inflammation. However, induction of peptic ulcer is a major adverse effect of NSAIDs that can promote erosion, hemorrhage, and mucosal perforation [47, 48]. Therefore, this pharmacological class has been widely used to establish animal models of the gastric ulcer [49]. Gastric complications caused by the administration of NSAIDs, such as piroxicam, occur due to inhibition of COX-1, the enzyme responsible for the production of protective prostaglandins. Consequently, the production and secretion of mucus and bicarbonate are affected by changes in the cellular integrity of the gastric mucosa, resulting in enhanced exposure to aggressive agents, such as acid and pepsin [50]. Prostaglandin also maintains mucosal blood flow and repairs and regulates mucosal cells. NSAIDs also increase mucosal hydrogen peroxide and hydroxyl ion concentrations by inhibiting gastric peroxide, which is responsible for oxidative mucosal damage [17].

As expected, in rats with piroxicam-induced ulcers and treated with water (Veh), severe damage to the gastric mucosa was observed. In contrast, pretreatment with AETo (30 or 300 mg/kg) revealed a gastroprotective effect, reducing the extent of the injured area. These effects were corroborated by an increase in the amount of PAS-stained mucin-like glycoproteins. Interestingly, in this glycoprotein assay, the CBX group (positive control) did not show a representative effect, suggesting a specific mechanism of AETo.

Further, intact gastric mucosal lining plays an important role in gastroprotection. The gastric mucosa may get damaged if microcirculation is disturbed. Therefore, improvement of blood flow in the gastric lining is essential to alleviate the gastric damage caused by ulcerogenic agents; hence, NO, which regulates blood flow, is a crucial factor [51]. A high level of NO in the gastric tissues of animals treated with *Taraxacum coreanum* Nakai increased the protection of the mucosal layer against the deterioration promoted by gastritis, partly by increasing the blood flow [52]. The AETo also presented ellagic acid, how a chemical constituent and this molecule exhibited antiulcer effect when evaluated in models of acute ulcer induced by ethanol or indomethacin and chronic ulcers induced by acetic acid. Thus, the gastroprotective mechanism of AETo probably also involves increased endogenous production of NO and reduced depletion of nonprotein sulfhydryl compounds, decreasing the secretion of TNF-*α* [53].

In addition, the production of mucus is an indicator of local gastric mucosal defense, which can be analyzed by PAS staining. Gastric mucus secretion is mainly composed of mucin, a macromolecular glycoprotein that accelerates epithelial recovery and forms a mucus layer that promotes tissue repair [54]. In this study, AETo significantly increased the amount of mucus in both ethanol- and piroxicam-induced ulcer groups compared to that in the CBX group, ensuring gastroprotection. Furthermore, high tannin content present in AETo (ellagic acid glucoside) is likely to be related to this protection mechanism because the interactions between tannins and biological macromolecules cause their precipitation over the mucosa, resulting in a mucoprotective barrier, an impenetrable layer to harmful agents [55].

Available data suggest that ROS plays a major role in tissue injury during the pathogenesis of various disorders of the digestive tract [56]. The production of free radicals occurs after exposure to certain types of stress, such as chronic use of ethanol and NSAIDs. Excess ROS causes lipid peroxidation, protein oxidation, DNA oxidation, and cell death, in addition to promoting inflammation [57, 58]. Antioxidant defense mechanisms regulate the production of free radicals and their metabolites to protect the gastric mucosa against damage caused by these oxidizing agents [59, 60].

It is well known that ROS can react with lipids to form lipid peroxides. As the major components of cell membranes are lipids, ROS may cause extensive damage. If the process is not neutralized by antioxidant molecules, lipid peroxidation can lead to cell death and/or apoptosis [61]. In the piroxicam-induced ulcer group, AETo (300 mg/kg) significantly decreased LOOH level compared to the vehicle group.

The reduced form of GSH is believed to act as the main intracellular antioxidant buffer with a multifaceted action against tissue oxidative agents. Reduced glutathione (GSH) is essential for the antioxidant defense mechanism; it protects the cells from oxygen reactive elements due to its ability to stabilize gastric mucus and maintain the integrity of the physiological system [62]. This tripeptide can be found in its reduced (GSSG) or oxidized (GSH) form, and it is used as a marker of the antioxidant capacity of biological systems. Being an electron donor, GSH is an essential cofactor for the reduction of H_2_O_2_ to H_2_O and O_2_ [59, 63]. In this study, pretreatment with AETo (300 mg/kg) prevented depletion of GSH levels in ethanol-treated rats, compared to the Veh group. This finding agrees with the results of the study by Park et al. [64] that evaluated the effect of an aqueous extract of *T. officinale* leaves on LPS-induced cells. In addition, glutathione S-transferase (GST) is involved in combating ROS, cell signaling, antiapoptotic activity, and anti- and proinflammatory responses. The main function of GST is to catalyze the conjugation of GSH with by-products derived from oxidative stress, allowing its elimination [65, 66]. AETo (30 and 300 mg/kg) showed an increase in GST activity in animals with piroxicam-induced ulcers, indicating protection of the gastric mucosa.

Other antioxidant defense enzyme systems that operate in conjunction with the enzymes mentioned above include superoxide dismutase (SOD), which promotes the dismutation of the superoxide radical (O_2_−) in hydrogen peroxide (H_2_O_2_) and oxygen (O_2_) and catalase (CAT), which converts H_2_O_2_ into H_2_O and O_2_ [67]. Furthermore, studies have shown that ethanol suppresses the mRNA expression and activities of antioxidant enzymes, such as SOD and CAT [51, 68]. Consequently, compared to the naive group, the groups treated with ethanol and piroxicam in the present study showed depletion of this enzyme. However, AETo treatment in both ulcer groups significantly increased the SOD activity, supporting the protection of the gastric mucosa.

CAT is an antioxidant enzyme that controls the accumulation of ROS generated through several metabolic processes. Inhibition of CAT activity leads to lipid peroxide formation by increasing the generation of hydroxyl radicals [69]. Thus, in the Veh group, the enzymatic activity of CAT was significantly decreased, while in the AETo and CBX groups, there was a significant increase in CAT activity.

Owing to its widespread use and pharmacological potential, several studies have been conducted to identify the constituents of *T. officinale*. These compounds have been identified in the subsequent studies and the present study mainly as phenolic acids and flavonoids [70–72]. Among these compounds, chicoric acid plays diverse roles as an antioxidant and in the prevention of inflammation [70, 73].

Myeloperoxidase (MPO) is an enzyme of the peroxidase subfamily. Although the complete biochemical mechanism of neutrophil degranulation is not yet clear, oxidative stress plays a key role in the release of MPO from these cells. Therefore, measurement of the MPO activity is conventionally used to infer leukocyte infiltration in many tissues and is considered an indirect marker of the presence of neutrophils. Such neutrophils migrate to the site of the inflammatory stimulus, and the method is based on the release of MPO to the injured tissue [74]. Thus, MPO is a biomarker of neutrophil infiltration, an important indicator of inflammation in the gastric mucosa [75]. As expected, both ethanol- and piroxicam-induced ulcer groups showed a high concentration of MPO in the rat stomach, while the AETo groups (30 and 300 mg/kg) decreased the activity of this enzyme, indicating a decrease in neutrophil recruitment. These results demonstrate a possible positive effect of AETo in reducing gastric inflammation and consequently gastric ulcers, which might be due to the presence of specific chemical compounds in AETo, including polyphenols and flavonoids, possessing inhibitory activities against MPO [76].

Ultrasonography is widely used in clinical practice. Recent technological advances have made it more sensitive to gastrointestinal tract diseases [77] in humans and rodents. On examination, gastric ulcer is suspected by the presence of focal wall thickening, associated or not with mucosal irregularity or depression [78]. The main indirect sign of the disease is the loss of well-defined histological layers. In an anatomical study by Tommaso et al. [25], based on the measurement of gastric wall thickness in healthy Wistar rats, it was possible to observe the inflammatory process in all animals subjected to injury induction. Using this technique in the present study, it was observed that the vehicle groups (ethanol and piroxicam) presented a greater thickness of the gastric wall, indicating the edema of the inflammatory process promoted by ulcerogenic agents, than that of the AETo or CBX groups. Thus, it can be proposed that AETo possesses gastroprotective effects through the modulation of polyphenolic compounds on inflammatory and oxidative stress parameters.

## 5. Conclusion

The aqueous extract *of T. officinale* exhibits a gastroprotective effect mediated by the reduction of oxidative stress parameters and modulation of the gastric inflammatory process. This effect may be due to the presence of many phenolic acids and flavonoids in the plant's leaves. In addition, this study proposes the use of ultrasonographic analyses as a new gastroprotective assessment tool in preclinical studies.

## Figures and Tables

**Table 1:** Phytochemical analysis of an aqueous extract of *Taraxacum officinale* (AETo) by ESI-IT-MSn^a^.

Compound	*m/z*	MS_2_	MS_3_ ⟶ MS^n^	Reference
	[M-H]^−^			
Gallic acid	169	125	−	[37]
Caffeic acid	179	135	−	[37]
Quinic acid	191	111, 173, 85, 127	−	[38]
Kaempferol	285	211, 127, 257, 151, 241	−	[38]
Ellagic acid	301	283, 272, 257	−	[38]
Dihydroxybenzoic acid glucoside	315	153, 109	−	[39]
Caffeoyl glucoside	377^b^	341	179, 161, 143, 149, 131	[37]
Kaempferol glucoside	447	285	267, 257, 241	[38]
Quercetin glucoside	463	301	179, 151	[38]
Ellagic acid glucoside	463	301	283, 272, 257	[38]
Chicoric acid	473	311, 293, 179, 149	−	[37]
Kaempferol rutinoside	593	285	285, 267, 257, 241	[37]
Quercetin-pentosyl-hexoside				
	595	**463,** 433	**301** ⟶ 151, 179	[38, 39]
Rutin				
	609	447, 301, 285	151, 179	[39]

Bold values indicate fragmentation sequence. ^a^Spectra recorded in negative mode. ^b^[M+Cl]−.

**Table 2:** Effects of aqueous extract from *Taraxacum officinale* (AETo) on oxidative parameters of ulcerated tissue.

Groups and treatments	GSH	LPO	SOD	CAT	GST
Naive	1340 ± 13.40	0.18 ± 0.007	46.03 ± 0.23	0.044 ± 0.03	0.32 ± 0.03
Ethanol group					
Vehicle	550.7 ± 4.22	0.17 ± 0.008	38.43 ± 0.47^bbb^	0.021 ± 0.02	0.31 ± 0.05
CBX	1030 ± 18.40	0.16 ± 0.005	43.78 ± 0.61^aaa^	0.191 ± 0.01^aaabbb^	0.73 ± 0.06
AETo (300)	1260 ± 17.70^aa^	0.16 ± 0.004	40.56 ± 0.54^a^	0.112 ± 0.02^aabbb^	0.18 ± 0.04
Piroxicam group					
Vehicle	850.6 ± 4.81	0.17 ± 0.003	13.55 ± 2.31^bbb^	0.033 ± 0.02	0.11 ± 0.01
CBX	980.8 ± 3.90	0.17 ± 0.007	23.35 ± .13^aa^	0.086 ± 0.02	0.26 ± 0.02
AETo (30)	780.9 ± 4.34	0.18 ± 0.006	21.41 ± 0.88^a^	0.131 ± 0.04	0.30 ± 0.02^a^
AETo (300)	580 ± 1.51	0.09 ± 0.005^aaa^	25.80 ± 0.78^aaa^	0.170 ± 0.07	0.65 ± 0.02^aaa^

*Note.* Carbenoxolone (CBX, 200 mg/kg); aqueous extract of *T. officinale* (AETo, 30 or 300 mg/kg); myeloperoxidase (MPO, mD.O/mg of protein); reduced glutathione (GSH, µg/mg of tissue); superoxide dismutase (SOD, U/mg of protein); catalase (CAT, µmol/min/mg of protein), and glutathione S-transferase (GST, µmol/min/mg of protein). Values are expressed as means ± SEM (*n* = 8). One-way ANOVA followed by Bonferroni's test. ^a^*p* < 0.05,  ^aa^*p* < 0.01,  ^aaa^*p* < 0.001 versus vehicle-treated group. ^bbb^*p* < 0.001 versus naive group.

## Data Availability

The articles, images, and analysis tables used to support the findings of this study are available from the corresponding author upon request.
